# Hepatitis as a Cause of Oedema

**Published:** 1915-01

**Authors:** 


**Hepatitis as a Cause of Oedema.** P. Le Damany, ''Rev. de Med.,'' July, 1914, has found oedemia, generally of the lower extremities, lumbar region and lower thorax but sometimes extending even to the arms, neck and face, in 17 of 36 cases. Most of the cases were alcoholic but tuberculous and syphilitic lesions were included. Renal and cardiac causes of oedema were excluded. The oedema generally ran parallel to ascites. He considers the explanation as metabolic rather than mechanic. (Note: Hanot's hypertrophic, alcoholic sclerosis of the liver is rather characteristically accompanied by marked ascites. In our experience, hypertrophic sclerosis is relatively rare and ascites sufficient to be detected by physical examination, even by auscultatory percussion, is comparatively rare in atrophic sclerosis, except in extreme cases usually found in hospital practice. Taking all cases of what may be termed hepatitis in the broad sense and therefore consisting mainly of contracted liver, ascites does not occur in more than 5%, although it might be found if all cases could be followed to the end. However, if ascites is demonstrable, more or less oedema is also usually demonstrable.)

